# Adverse fibrosis remodeling and aortopulmonary collateral flow are associated with poor Fontan outcomes

**DOI:** 10.1186/s12968-021-00782-9

**Published:** 2021-11-15

**Authors:** Andrea Pisesky, Marjolein J. E. Reichert, Charlotte de Lange, Mike Seed, Shi-Joon Yoo, Christopher Z. Lam, Lars Grosse-Wortmann

**Affiliations:** 1 Department of Paediatrics, Division of Cardiology, The Hospital for Sick Children, University of Toronto, Labatt Family Heart Center, 555 University Avenue, Toronto, ON M5G 1X8 Canada; 2 Division of Radiology and Nuclear Medicine, Pediatric section, Rikshospitalet, Oslo University Hospital, Oslo, Norway; 3 Department of Diagnostic Imaging, The Hospital for Sick Children, University of Toronto, Toronto, ON Canada; 4 Department of Pediatrics, Doernbecher Children’s Hospital, Oregon Health and Science University, Portland, OR USA

**Keywords:** Congenital heart disease, Fontan, Heart failure, Fibrosis, Magnetic resonance imaging

## Abstract

**Background:**

The extent and significance in of cardiac remodeling in Fontan patients are unclear and were the subject of this study.

**Methods:**

This retrospective cohort study compared cardiovascular magnetic resonance (CMR) imaging markers of cardiac function, myocardial fibrosis, and hemodynamics in young Fontan patients to controls.

**Results:**

Fifty-five Fontan patients and 44 healthy controls were included (median age 14 years (range 7–17 years) vs 13 years (range 4–14 years), p = 0.057). Fontan patients had a higher indexed end-diastolic ventricular volume (EDVI 129 ml/m^2^ vs 93 ml/m^2^, p < 0.001), and lower ejection fraction (EF 45% vs 58%, p < 0.001), circumferential (CS − 23.5% vs − 30.8%, p < 0.001), radial (6.4% vs 8.2%, p < 0.001), and longitudinal strain (− 13.3% vs − 24.8%, p < 0.001). Compared to healthy controls, Fontan patients had higher extracellular volume fraction (ECV) (26.3% vs 20.6%, p < 0.001) and native T1 (1041 ms vs 986 ms, p < 0.001). Patients with a dominant right ventricle demonstrated larger ventricles (EDVI 146 ml/m^2^ vs 120 ml/m^2^, p = 0.03), lower EF (41% vs 47%, p = 0.008), worse CS (− 20.1% vs − 25.6%, p = 0.003), and a trend towards higher ECV (28.3% versus 24.1%, p = 0.09). Worse EF and CS correlated with longer cumulative bypass (R = − 0.36, p = 0.003 and R = 0.46, p < 0.001), cross-clamp (R = − 0.41, p = 0.001 and R = 0.40, p = 0.003) and circulatory arrest times (R = − 0.42, p < 0.001 and R = 0.27, p = 0.03). T1 correlated with aortopulmonary collateral (APC) flow (R = 0.36, p = 0.009) which, in the linear regression model, was independent of ventricular morphology (p = 0.9) and EDVI (p = 0.2). The composite outcome (cardiac readmission, cardiac reintervention, Fontan failure or any clinically significant arrhythmia) was associated with increased native T1 (1063 ms vs 1026 ms, p = 0.029) and EDVI (146 ml/m^2^ vs 118 ml/m^2^, p = 0.013), as well as decreased EF (42% vs 46%, p = 0.045) and worse CS (− 22% vs − 25%, p = 0.029). APC flow (HR 5.5 CI 1.9–16.2, p = 0.002) was independently associated with the composite outcome, independent of ventricular morphology (HR 0.71 CI 0.30–1.69 p = 0.44) and T1 (HR1.006 CI 1.0–1.13, p = 0.07).

**Conclusions:**

Pediatric Fontan patients have ventricular dysfunction, altered myocardial mechanics and increased fibrotic remodeling. Cumulative exposure to cardiopulmonary bypass and increased aortopulmonary collateral flow are associated with myocardial dysfunction and fibrosis. Cardiac dysfunction, fibrosis, and collateral flow are associated with adverse outcomes.

**Supplementary Information:**

The online version contains supplementary material available at 10.1186/s12968-021-00782-9.

## Background

By early adulthood, up to 50% of patients with a Fontan circulation are in heart failure, [1, 2] a common cause of late death in this population [2–5]. There is growing evidence that functionally univentricular hearts undergo accelerated remodeling, possibly contributing to decreased contractility, diastolic dysfunction and deteriorating functional status [6, 7]. Diffuse myocardial fibrosis is an increasingly recognized mechanism of ventricular dysfunction [8, 9]. However, little is known about its prevalence, extent, and impact on myocardial function in the Fontan population [10–13].

## Methods

### Study aim and hypothesis

The aims of the present study were to investigate the degree of functional and fibrotic myocardial remodeling present in young Fontan patients, to unveil candidate etiologies for alterations in structure and contractility, and to describe their potential functional impact. We hypothesized that ventricular dysfunction and fibrotic myocardial remodelling are enhanced in the subgroup of Fontan patients with a dominant right ventricle (DRV).

### Study design

Fontan patients and control subjects who had received cardiovascular magnetic resonance (CMR) imaging between April 2014 and March 2018 were included in this retrospective cohort study. The study setting is a tertiary care hospital. If more than one CMR had been performed in the same patient, only the last CMR was included. Scans were excluded if non-diagnostic. Further, Fontan patients with two nearly equal sized ventricles were excluded to allow for a comparison between patients with DRV and dominant left ventricle (DLV). Patients referred for coronary artery imaging because of non-specific chest pain or for a family history of arrhythmogenic right ventricular cardiomyopathy were included as controls if their work-up, including CMR, was negative. Twenty-one Fontan patients and 24 healthy control subjects were previously reported [13]. The prior pilot study characterized CMR markers of fibrosis, while the present article examines correlations with functional data and associations with clinical outcomes.

### Surgical and clinical data

Demographic, surgical, and clinical data were retrieved from the patients’ medical records. Results from cardiac catheterization, echocardiography, and cardiopulmonary exercise testing were collected if the test had occurred within 12 months of CMR, without an interim intervention. ‘Failing Fontan circulation’ was defined by the presence of plastic bronchitis, protein-losing enteropathy, anasarca, or surgical take-down of the Fontan within 6 months of CMR. A composite outcome was used to assess the predictive value of CMR and included any of the following at or after the CMR, up until study completion: readmission for cardiac indication, cardiac reintervention, Fontan failure or any clinically significant arrhythmia. Of note, the former patients with Fontan failure were also included in the composite outcome. Significant atrioventricular valvar regurgitation (AVVR) was defined as at least moderate insufficiency by echocardiography.

### Cardiovascular Magnetic Resonance

All examinations were conducted on a 1.5 T CMR system (Avanto, Siemens Healthineers, Erlangen, Germany). The protocol included balanced steady-state free precession short-axis cine stacks for ventricular volumetry, as previously described [8]. Phase contrast flow velocity mapping for blood flow volumes was obtained in the thoracic arteries and veins as well as the Fontan circuit [14]. A phase-sensitive inversion recovery sequence was used for late gadolinium enhancement (LGE) imaging ten minutes after injection of 0.2 mmol/kg gadobenate dimeglumine (‘Multihance’, Bracco S.p.A., Milan, Italy) or gadobuturol (‘Gadavist’, Bayer Healthcare, Berlin, Germany). Myocardial T1 values were obtained using a Modified Look-Locker inversion recovery (MOLLI) sequence at a midventricular short-axis location before and 15 min after the administration of gadolinium, as detailed [13, 15].

### Ventricular volumetry, blood flows, and T1 relaxometry analysis

A single observer (MR) with several months of focused training and under the supervision of a single senior reader (LGW) for each case analyzed short axis cine stacks for ventricular volumes using commercially available software Qmass (version 8.1, Medis Medical Imaging Systems, Leiden, the Netherlands). In patients with a ventricular septal defect or a communication of the hypoplastic ventricle with the neo-aorta, the smaller chamber was included in the single ventricular contours. Blood flow volumes were quantified using Qflow (version 8.1, Medis Medical Imaging Systems). Aortopulmonary collateral (APC) flow was calculated as the difference between pulmonary venous and arterial flows, indexed to body surface area.

**Fig. 1:**
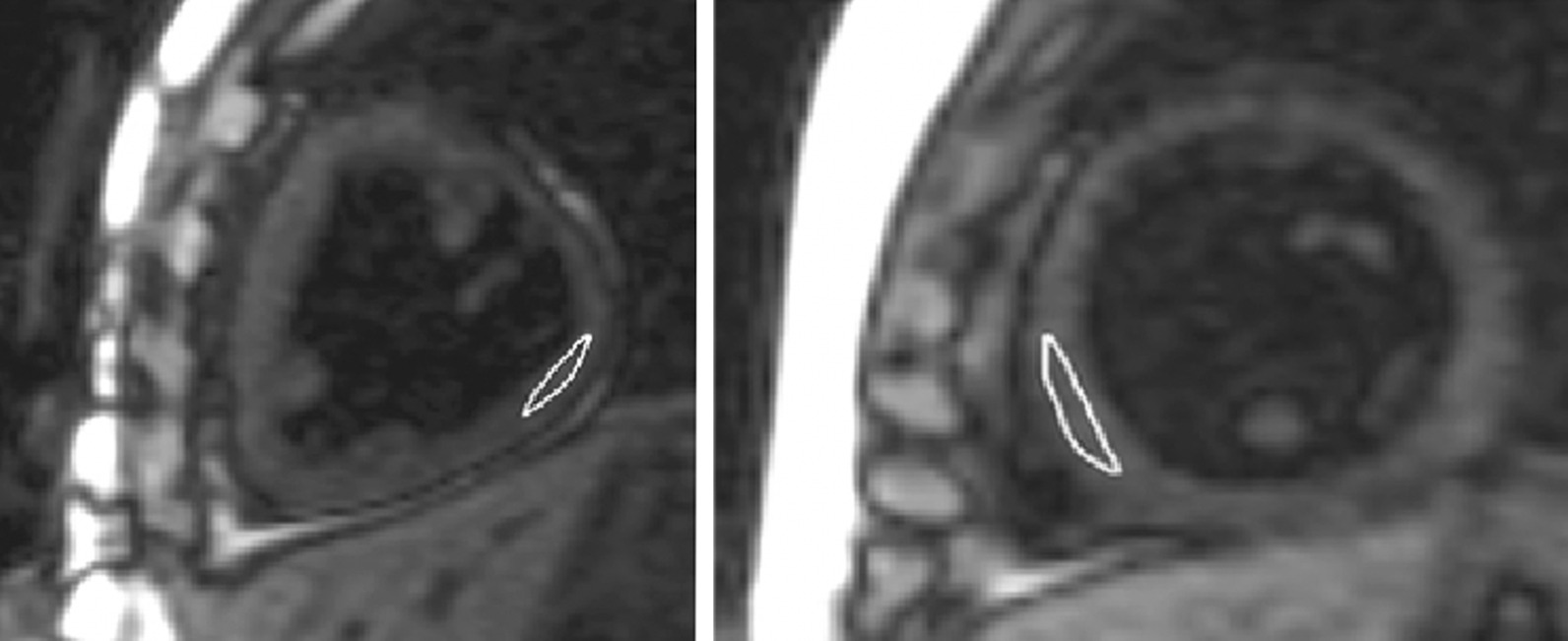
Frame from the modified Look-Locker inversion recovery acquisition in short axis orientation. Myocardial T1 measurements in Fontan patients with a dominant right (right) and a left ventricle (left). Regions of interest were drawn in the free wall of the dominant ventricle

T1 relaxometry analysis was performed by a single observer (CdL) with 10 years of CMR experience on the online motion-corrected source images, using’Qmap 2.2.36’ (Medis Medical Imaging Systems). Regions of interest (ROIs) were drawn in the freewall of the dominant ventricle, propagated to each of the eight source images and adjusted for motion (Fig. 1). In controls, T1 was measured in the entire left ventricular myocardium. Only the central third of the myocardium was included in order to avoid partial-voluming with blood or epicardial fat. Areas of LGE were excluded from T1 ROIs. Extracellular volume (ECV) was calculated with patient’s hematocrit and the pre- and post-contrast T1 values [16]. To assess the reproducibility of myocardial T1 measurements, a random sample of 20 patients was re-measured by a second observer (MR) after several months of full-time training in postprocessing. For intra-observer variability the same observer re-measured T1 and ECV on the same sample. All measurements were completed under the supervision of a CMR staff (LGW) with 15 years of CMR experience.

### Strain, wall and fiber stress

Ventricular myocardial strain was obtained using CMR feature tracking from the steady state free precession cine images Qstrain (version 2.0, Medis Medical Imaging Systems), as previously described [13, 17]. Segments including a ventricular septal defect or the ventricular outflow tract were excluded. Peak longitudinal strain (LS) of the ventricular inferior wall was retrieved from the vertical long axis. Global ventricular strain was computed by averaging segmental values at all ventricular levels. The standard deviation of the times to peak circumferential strain (CS) of all midventricular short axis segments was calculated as a metric for intraventricular dyssynchrony [18, 19]. End-systolic wall-stress (ESWS) and end-systolic fiber stress (ESFS) were computed using published equations [7, 20]. Ventricular arterial coupling (VAC) ratio was calculated non-invasively, by dividing the arterial elastance (Ea) by the end-systolic ventricular elastance (Ees), according to previously published formulas [6] (Additional file 1).

### Statistical analysis

Continuous variables are presented as means ± standard deviations if normally distributed, otherwise as medians and ranges. For normally distributed continuous variables, two-tailed independent sample t-tests were used in conjunction with Levene’s Test of Equality of Variances. For non-normally distributed continuous variables Wilcox rank sum test was used. Univariate and multivariable regressions were performed to explore presence of linear relationships between measurements in the overall cohort. Correlations were assessed using Pearson’s correlation coefficient analysis for normally distributed continuous variables and Spearman rank-order test for non-normally distributed data. Intra- and interobserver variability of T1 and ECV was analyzed using Bland–Altman plots. P-values < 0.05 were considered statistically significant (Additional file 1: Fig S1A–D). Cox regression analysis predicting the composite outcome was complete using variables significant in univariate analysis. Statistical analyses were performed using SPSS (version 25, Statistical Package for the Social Sciences, International Business Machines, Inc., Armonk, New York, USA). Sample size calculations were not performed for this retrospective study, but requirements for statistical power were observed for all tests.

## Results

### Demographic, surgical, and clinical data

**Fig. 2:**
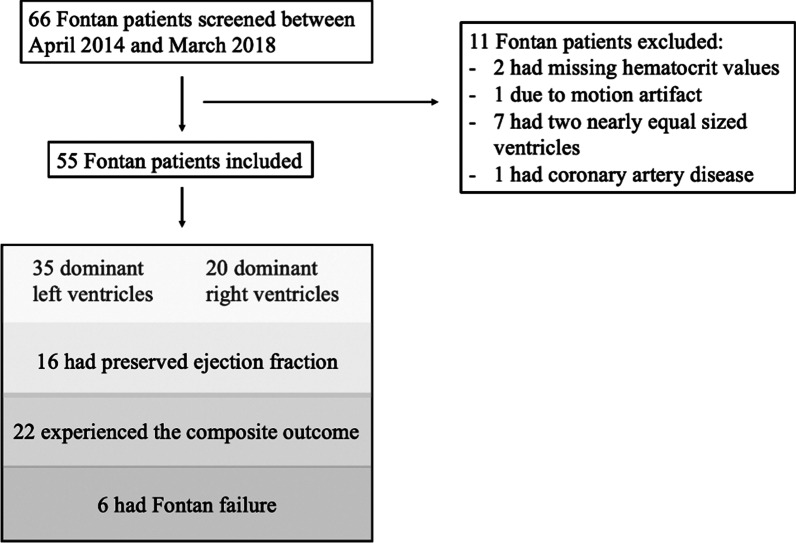
Study flow diagram

Sixty-six single ventricle patients were screened, of which 11 were excluded: two patients were missing hematocrit values precluding computation of ECV, one had non-diagnostic images due to motion artifacts, seven had two nearly equal-sized ventricles, and one had concomitant Kawasaki disease with stenosis of the left coronary artery (Fig. 2). The 44 controls (24 of 41 (59%) male) were a mean of 3.9 years older (p = 0.35 and 24.4 kg heavier (p = 0.56) than the Fontan patients (Table 1). Of the 55 Fontan cohort, 35 patients (64%) had a DLV and 20 (36%) a DRV. The most common diagnosis in the DLV and DRV cohorts were tricuspid atresia (10 of 20 (50%)) and hypoplastic left heart syndrome (33 of 35 (94%)), respectively. Seventeen patients of the DRV cohort (85%) and 18 of the DLV cohort (51%) had neonatal surgery (p = 0.013). The DRV cohort had longer cumulative bypass (p = 0.011), aortic cross clamp (p = 0.027), and deep hypothermic circulatory arrest times (p = 0.01, Table 1).

**Table 1:** Clinical characteristics of control and Fontan cohort, with subgroups according to ventricular morphology

	Control N = 41	Fontan N = 55	P value	DRV N = 20	DLV N = 35	P value
Demographics						
Gender (male)	59%	58%	0.93	50%	69%	0.35
Median age (years)	14 (7–17)	13 (4–14)	0.057	12.3 (3.6–16.9)	13.5 (4–18)	0.35
Surgical data						
Median age at BCPC (months)		6 (2–26)		6 (2–26)	6 (2–10)	0.09
Median age at Fontan (months)		41 (16–82)		42 (18–58)	41 (16–82)	0.80
Cumulative bypass time (min)		229 ± 122		283 ± 120	199 ± 114	0.01
Cumulative cross clamp time (min)		61 ± 62		85 ± 53	48 ± 63	0.03
Cumulative DHCA time (min)		6 ± 12		12 ± 14	2 ± 10	0.01
Clinical information						
Height (cm)	164 (123–190)	151 (99–183)	< 0.001	151 (99–173)	152 (103–183)	0.54
Weight (kg)	60 (22–125)	34 (15–146)	< 0.001	38 (15–146)	43 (16–89)	0.56
Heart rate (bpm)	73 (46–105)	81 (57–145)	0.005	85 (63–145)	79 (57–110)	0.03
Saturations (%)		95 (78–98)		95 (79–98)	95 (78–98)	0.87
Systolic BP percentile		64 (3–100)		60 (10–98)	64 (3–100)	0.82
Diastolic BP percentile		39 (8–100)		30 (18–97)	43 (8–100)	0.31
Hematocrit	0.43 ± 0.04	0.44 ± 0.04	0.29	0.44 ± 0.04	0.46 ± 0.05	0.21
Cardiac catheterization						
Median interval CMR to Cath (days)		0 (0–206)		0 (0–206)	0 (0–133)	0.44
SVEDP (mmHg)		7 ± 2		6 ± 2	8 ± 2	0.27
CVP (mmHg)		10 ± 12		11 ± 2	10 ± 2	0.62
MAP (mmHg)		4 ± 2		5 ± 1	6 ± 2	0.50
Echocardiography						
Median interval CMR to echo (days)		64 (1–365)		67 (1–365)	60 (2–361)	0.57
Moderate to severe AVVR		31% (16)		45% (9)	23% (7)	0.13
Moderate to severe AR		8% (4)		5% (1)	10% (3)	0.50
Cardiopulmonary exercise testing^a^						
Median interval CMR to CPET (days)		88 (0–365)		53 (16–343)	92 (0–341)	0.97
VO_2_max (%predicted)		75.5 ± 12.1		74.8 ± 10.9	76.3 ± 13.2	0.78
Work-load (%predicted)		81.2 ± 16.1		89.6 ± 11.5	82.0 ± 19.0	0.74
Anaerobic threshold (% predicted)		80.9 ± 17.1		84.0 ± 17.1	79.3 ± 18.1	0.56

^a^CPET within one year of CMR was only available in 28 Fontan patients, 19 with a DLV and 9 with a DRV

AR, aortic regurgitation; AVVR, atrioventricular valvar regurgitation; BCPC, Bidirectional Cavopulmonary Connection; BP, blood pressure; BSA, body surface area; Cath, cardiac catheterization; CMR, cardiovascular magnetic resonance; CPET, cardiopulmonary exercise testing; CVP, central venous pressure; DHCA, deep hypothermic circulatory arrest; DLV, dominant left ventricle; DRV, dominant right ventricle; MAP, mean atrial pressure; SVEDP, single ventricle end diastolic pressure; VO_2max_, maximum rate of oxygen consumption

### Cardiac catheterization, echocardiography, and cardiopulmonary exercise testing

Twenty-one of the 55 Fontan patients (38%) had undergone cardiac catheterization within one year of CMR with a median interval of zero days (range 0–206 days). There was no difference in the central venous, atrial or ventricular end-diastolic pressure between DLV and DRV patients (Table 1). Echocardiograms within one year of the CMR were available in 93%, within a median of 64 days from CMR (range 1–365 days).

Cardiopulmonary exercise test results were available within one year of CMR in 28 of the 55 Fontan patients (51%), at a median of 87 days (range 0–365 days). There were no differences in the maximum oxygen consumption, anaerobic threshold or maximum work-load between DLV and DRV patients or between those with and without significant AVVR. There was no correlation between exercise parameters and myocardial strain, ejection fraction (EF), ventricular volumes, fibrosis markers, ESFS, or ESWS.

### Ventricular volumes and function, myocardial contractility

Ventricular volumes and myocardial function indices are provided in Table 2 for the entire cohort as well as stratified by ventricular dominance and by the presence of the composite outcome (Table 3). Bland Altman plots revealed good inter-observer and intra-observer agreement for T1 (bias − 1 ± 32 ms and limits of agreement (LOA)-64 to 63 ms; bias − 6 ± 12 ms and LOA − 18 to 30 ms, respectively) and ECV (bias 0.9 ± 1.5% and LOA − 2.0 to 3.8%; bias − 0.8 ± 2.1% and LOA − 4.9 to 3.3%, respectively) measurements (Additional file 1: Figs S1–S4). Compared to controls, the Fontan cohort had higher end-diastolic volume index (EDVI) and end-systolic volume index (ESVI) as well as reduced EF, global CS, radial strain (RS), and LS (p < 0.001 for all). Even patients with relatively preserved systolic function, defined as EF ≥ 50% (16 of 55 (29%)), had lower CS (− 28.2 ± 2.9% versus − 30.9 ± 3.6%, p = 0.008), radial strain (RS) (7.0 ± 1.3% versus 8.2 ± 1.1%, p = 0.003), and LS (− 16.6 ± 10.9% versus − 24.9 ± 4.4%, p = 0.041) as compared to controls. On average, patients with DRV had larger EDVI and ESVI (p = 0.03 and p = 0.02, respectively) and lower EF and CS (p = 0.008 and p = 0.003, respectively) than DLV. Sixteen of 52 Fontan patients (31%) had significant AVVR, a subgroup displaying decreased global CS (− 19.8 ± 5.8% versus − 25.1 ± 4.9%, p = 0.003), with no difference in EF (43 ± 9% versus 45 ± 8%, p = 0.3). There was a trend towards larger EDVI in patients with significant AVVR (144 ± 54 ml/m^2^ versus 122 ± 36 ml/m^2^, p = 0.08).

**Table 2:** Cardiovascular magnetic resonance results for ventricular volumetry, myocardial mechanics by feature tracking, myocardial scarring and fibrosis, and cardiovascular efficiency and stress

	Healthy control (SD) N = 41	Fontan (SD) N = 55	P value	DRV (SD) n = 20	DLV (SD) N = 35	P value
Volumetry and flows						
Mass indexed (g/m^2^)	56 (13)	72 (22)	< 0.001	76 (26)	70 (20)	0.32
Mass/EDV	0.60 (0.11)	0.57 (0.10)	0.14	0.53 (0.08)	0.59 (0.10)	0.02
EDVI (mL/m^2^)	93 (13)	129 (42)	< 0.001	146 (50)	120 (35)	0.03
ESVI (mL/m^2^)	39 (7)	73 (32)	< 0.001	89 (41)	64 (21)	0.02
SVSVI (mL/m^2^)	54 (9)	56 (17)	0.51	56 (13)	56 (19)	0.88
SVEF (%)	58 (5)	45 (8)	< 0.001	41 (8)	47 (8)	0.008
APC flow (L/m^2^)		0.64 (0.45)		0.67 (0.51)	0.61 (0.41)	0.65
Feature tracking						
Longitudinal strain (%)	− 24.8 (4.3)	− 13.3 (9.5)	< 0.001	− 13.1 (10.0)	− 13.4 (0.8)	0.96
Radial strain (%)	8.2 (1.1)	6.4 (1.4)	< 0.001	6.5 (1.6)	6.4 (1.4)	0.81
Circumferential strain (%)	− 30.8 (3.6)	− 23.5 (5.7)	< 0.001	− 20.1 (6.3)	− 25.6 (4.2)	0.003
STD-T2P	1.4 (0.9)	1.8 (0.9)	0.043	2.1 (0.8)	1.7 (1.0)	0.13
Myocardial fibrosis and scarring						
ECV free wall (%)	20.6 (2.6)	26.3 (6.7)	< 0.001	28.3 (8.7)	25.1 (5.01)	0.09
Native T1 free wall (ms)	986 (49)	1041 (62)	< 0.001	1056 (55)	1032 (65)	0.17
LGE prevalence (%)		15 (8)		10 (2)	17 (6)	0.56
Coupling ratio, wall and fiber stress						
VAC ratio	0.7 (0.1)	1.3 (0.5)	< 0.001	1.6 (0.6)	1.2 (0.4)	0.01
ESWS (kPA)		18.1 (3.9)		19.6 (4.4)	17.0 (3.5)	0.02
ESFS (kPA)		12.1 (2.0)		12.1 (1.4)	12.3 (2.0)	0.79

APC, aortopulmonary collateral; DLV, dominant left ventricle; DRV, dominant right ventricle; ECV, extracellular volume; EDVI,end diastolic volume indexed; ESFS, end systolic fiber stress; ESVI, end systolic volume indexed; ESWS, end systolic wall stress; LGE, late gadolinium enhancement; STD-T2P, standard deviation of the times to peak CS of all midventricular short axis segments; SVEF, systemic ventricle ejection fraction; SVSVI, systemic ventricle strove volume indexed; VAC, ventricular arterial coupling

Wilcoxon rank-sum test for continuous variables, and Fisher's exact test for categorical variables

**Table 3:** Clinical characteristics of Fontan patients with and those without the composite outcome

	Composite outcome N = 22	No composite outcome N = 33	P value
Demographics			
Gender (male)	11 (50%)	21 (64%)	0.315
Median age (years)	10 (4–16)	14 (5–18)	0.35
DLV	11 (50%)	24 (73%)	0.086
Surgical data			
Median age at BCPC (months)	5 (2–8)	6 (2–12)	0.26
Median age at Fontan (months)	37 (29–61)	40 (18–56)	0.52
Cumulative bypass time (min)	252 ± 129	213 ± 117	0.256
Cumulative cross clamp time (min)	81 ± 59	48 ± 61	0.055
Cumulative DHCA time (min)	10 ± 14	3 ± 10	0.063
Clinical information			
Height (cm)	136 (100–183)	154 (99–180)	0.54
Weight (kg)	33 (15–62)	47 (21–146)	0.56
Heart rate (bpm)	85 (61–145)	78 (57–105)	0.025
Saturations (%)	94 (78–98)	95 (91–98)	0.87
Systolic BP percentile	68 (13–100)	63 (3–100)	0.82
Diastolic BP percentile	40 (11–100)	37 (8–99)	0.31
Hematocrit	0.44 ± 0.04	0.45 ± 0.04	0.964
Cardiac catheterization			
Median interval cath to MRI (days)	0 (0–206)	0 (0–103)	0.617
SVEDP (mmHg)	7 ± 3	9 ± 3	0.232
CVP (mmHg)	10 ± 1	11 ± 2	0.406
MAP (mmHg)	5 ± 1	7 ± 4	0.256
Echocardiography			
Median interval CMR to echo (days)	52 (6–226)	124 (2–351)	0.57
Moderate to severe AVVR	8 (36%)	8 (24%)	0.332
Moderate to severe AR	0	4 (12%)	0.090
Cardiopulmonary exercise testing			
Median interval CMR to CPET (days)	27 (0–337)	145 (0–343)	0.97
VO_2_ max (% predicted)	78.2 ± 9.7	75.9 ± 16.8	0.70
Work-load (% predicted)	77.1 ± 17.2	85.1 ± 14.1	0.211
Anaerobic threshold (% predicted)	77.2 ± 16.4	77.1 ± 16.4	0.104

AR, aortic regurgitation; AVVR, atrioventricular valvar regurgitation; BCPC, Bidirectional Cavopulmonary Connection; BP, blood pressure; BSA, body surface area; Cath, cardiac catheterization; CPET, cardiopulmonary exercise testing; CVP, central venous pressure; DHCA, deep hypothermic circulatory arrest; DLV, Dominant left ventricle; DRV, Dominant right ventricle; MAP, mean atrial pressure; SVEDP, single ventricle end diastolic pressure; VO_2 max_, maximum rate of oxygen consumption

Worse EF and CS correlated with cumulative bypass (R = − 0.36, p = 0.003 and R = 0.46, p < 0.001, respectively), aortic cross clamp (R = − 0.41, p = 0.001 and R = 0.40, p 0.003, respectively) and circulatory arrest (R = − 0.42, p = 0.001 and R = 0.27, p = 0.03, respectively) times. EDVI correlated with APC flow (R = 0.45, p < 0.001). There was a correlation between ECV and EDVI (R = 0.46, p < 0.001, respectively) that, in linear regression, was independent of APC flow (B = − 0.071, p = 0.63) and ventricular morphology (B = 1.55, p = 0.62). As compared to controls, the Fontan cohort demonstrated evidence of intraventricular dyssynchrony with an increased standard deviation of the times to peak CS (p = 0.04).

### Ventricular-arterial coupling, myocardial elastance, fiber and wall stress

The VAC ratio was higher in Fontan patients as compared to controls subjects (p < 0.001) with a greater range and standard deviation. The DRV group demonstrated a higher VAC ratio (p = 0.015), Ees (p = 0.019) and ESWS (p = 0.006) as compared to the DLV group (Table 2). Patients with a failing Fontan circulation or having reached the composite outcome had higher VAC ratios as compared to the remainder of the patient cohort (1.9 ± 0.6 vs 1.3 ± 0.4 p = 0.002 and 1.5 ± 0.5 vs 1.2 ± 0.4, p = 0.043, respectively) and no differences in fibre or wall stress.

There were no correlations between the haemodynamic or exercise data and wall or fibre stress, or VAC. Ees correlated with cumulative bypass (R − 0.36, p = 0.009), aortic cross clamp (R − 0.44 p = 0.001) and circulatory arrest times (R − 0.43 p = 0.001). VAC, ESWS and ESFS correlated inversely with EF (R = − 0.96 p < 0.001, R = − 0.56, p < 0.001, and R = − 0.54 p < 0.001, respectively) and correlated positively with CS (R = 0.83 p < 0.001, R 0.57, p < 0.001 and R = 0.30, p = 0.02, respectively).

### Myocardial fibrosis and scarring

**Fig. 3:**
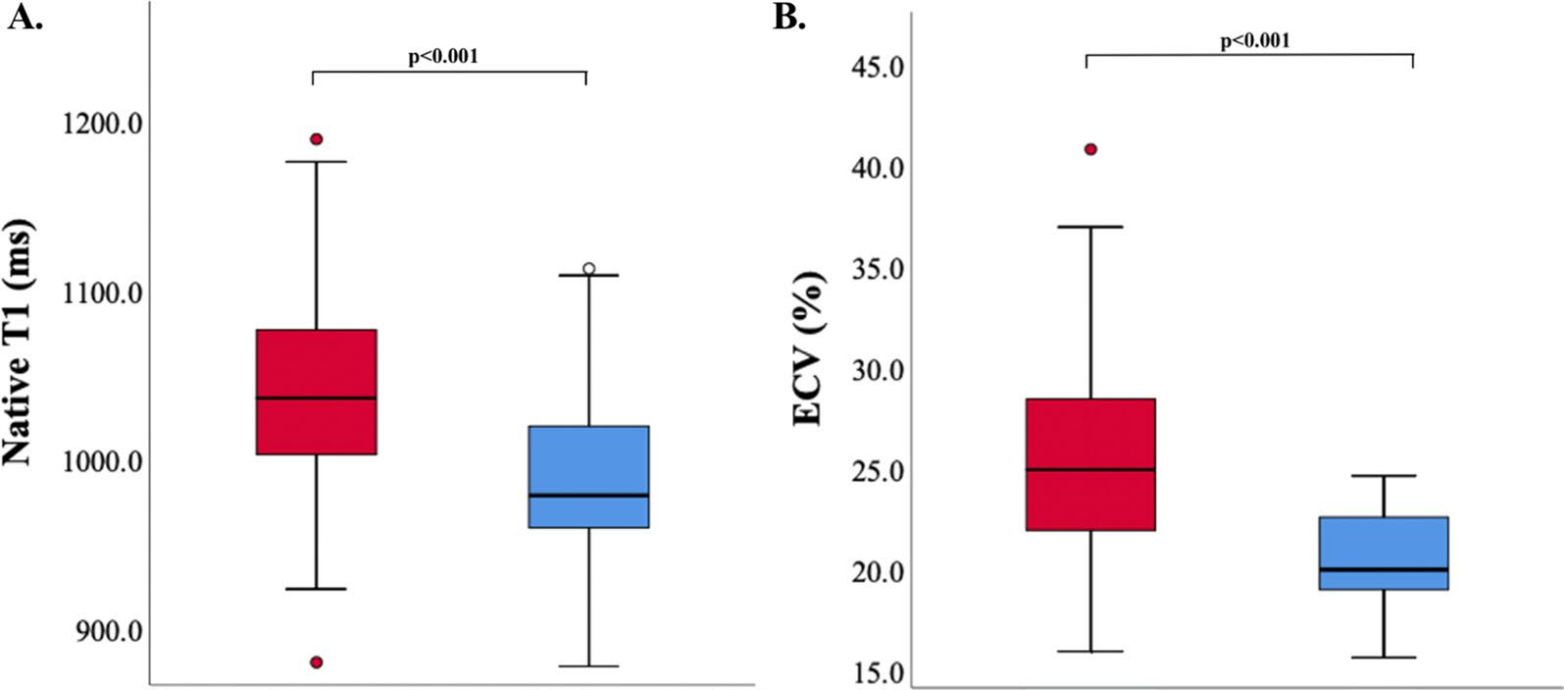
Comparison of native T1 and extracellular volume fraction (ECV) values between healthy controls subjects and Fontan patients. **A** Myocardial T1 measurements in healthy control subjects (red) and Fontan patients (blue); **B** ECV measurements in control subjects (red) and Fontan patients (blue)

LGE was present in five if the 55 Fontan patients (10%) and in no controls. Myocardial T1 and ECV measurements demonstrated adequate reproducibility (Additional file 1: Fig S1A–D). The Fontan cohort had higher ECV and T1 as compared to controls (Fig. 3A and B; p < 0.001). Excluding Fontan patients with failure, the Fontan cohort still demonstrated higher ECV and native T1 as compared to controls (25.4 ± 4.9 vs 20.6 ± 2.6, p < 0.001 and 1034 ± 58 ms vs 986 ± 49 ms, p < 0.001, respectively). Even Fontan patients with preserved EF had higher ECV and T1 as compared to controls (24.2 ± 5.2% versus 20.6 ± 2.6%, p = 0.021 and 1026 ± 60 ms versus 987 ± 50 ms, p = 0.018, respectively). There was a trend towards higher ECV in patients with a DRV as compared to patients with a DLV (p = 0.093). Patients with moderate to severe AVVR (n = 16 of 52) did not have increased T1 (1049 ± 48 ms versus 1037 ± 68 ms, p = 0.5) or ECV (27 ± 7.4% versus 26 ± 4.5%, p = 0.7). Worse RS, CS and LS correlated with T1 (R = − 0.50, p < 0.001; R = 0.35, p < 0.001; and R = 0.33, p = 0.005, respectively) and ECV (R = − 0.42, p < 0.001; R = 0.50, p < 0.001; and R = 0.48, p = 0.001, respectively).

We found no correlation between cumulative bypass, cross clamp or circulatory arrest time and markers of fibrosis. T1 correlated with APC flow (R = 0.36, p = 0.005) which, in a linear regression model, was independent of ventricular morphology (Beta = − 0.021, p = 0.889) and EDVi (Beta = 0.177, p = 0.188).

### Outcomes

We investigated the following two outcome surrogates: clinical Fontan failure concurrent to CMR and the composite outcome. Within six months of CMR, six of 55 Fontan patients (9%), four with a DRV and two with a DLV, had a failing Fontan physiology. The six patients with Fontan failure had decreased EF (36 ± 8% versus 46 ± 8%, p = 0.005), global CS (− 18.1 ± 5.6% versus − 24.3 ± 5.3%, p = 0.01) and RS (5.0 ± 1.8% versus 6.6 ± 1.3%, p = 0.009) and higher ECV (34.2 ± 13.2% versus 25.3 ± 4.8%, p = 0.002) and T1 (1103 ± 71 ms versus 1033 ± 57 ms, p = 0.008). Twenty-two of the 55 Fontan patients (40%) experienced the composite outcome (Table 3): cardiac reintervention n = 17, clinically significant arrhythmias n = 5, clinical Fontan failure n = 11 and cardiac readmission n = 9. Twelve Fontan patients experienced more than one composite outcome. Patients having experienced the composite outcome had lower EF (p = 0.045) and CS (p = 0.029), and increased EDVI (p = 0.013), ventricular arterial coupling (VAC) ratio (p = 0.043), APC flow (p = 0.027) and T1 (p = 0.029 Table 4). There was no difference in the invasive hemodynamics or exercise capacity between those with and those without the composite outcome or Fontan failure (Table 3).

**Table 4:** Cardiovascular magnetic resonance results for Fontan patients with and those without the composite outcome

	Composite outcomes N = 22	No composite outcome N = 33	P Value
Volumetry and flows			
Mass indexed (g/m^2^)	79.5 (22.8)	67.0 (20.6)	0.039
Mass/volume	0.55 (0.09)	0.57 (0.11)	0.397
EDVI (mL/m^2^)	146.2 (50.0)	117.5 (36.2)	0.013
ESVI (mL/m^2^)	87.2 (37.9)	63.6 (23.8)	0.006
SVSVI (mL/m^2^)	58.9 (18.2)	53.9 (18.2)	0.239
SVEF (%)	41.9 (8.9)	46.4 (7.5)	0.045
APC flow (L/m^2^)	0.81 (0.50)	0.51 (0.38)	0.017
Feature tracking			
Longitudinal strain (%)	− 12.4 (6.3)	− 15.4 (6.5)	0.299
Radial strain (%)	6.1 (1.6)	6.6 (1.3)	0.264
Circumferential strain (%)	− 21.5 (6.8)	− 25.0 (4.2)	0.029
STD-T2P	2.0 (0.9)	1.7 (0.9)	0.287
Myocardial fibrosis and scarring			
ECV freewall (%)	25.1 (4.4)	28.1 (8.9)	0.098
Native T1 freewall (ms)	1063 (62)	1026 (59)	0.029
LGE prevalence (%)	2 (9%)	6 (18%)	0.153
Coupling and wall and fiber stress			
VAC ratio	1.5 (0.6)	1.2 (0.4)	0.043
ESWS (kPA)	19.3 (4.1)	17.4 (3.6)	0.084
ESFS (kPA)	12.6 (1.9)	12.0 (1.7)	0.225

APC, aortopulmonary collateral; DLV, dominant left ventricle; DRV, dominant right ventricle; ECV, extracellular volume; EDVI,end diastolic volume indexed; ESFS, end systolic fiber stress; ESVI, end systolic volume indexed; ESWS, end systolic wall stress; LGE, late gadolinium enhancement; STD-T2P, standard deviation of the times to peak CS of all midventricular short axis segments; SVEF, systemic ventricle ejection fraction; SVSVI, systemic ventricle strove volume indexed; VAC, ventricular arterial coupling

Wilcoxon rank-sum test for continuous variables, and Fisher's exact test for categorical variables

The Cox regression model was restricted to clinical variables significantly associated with the composite outcome by univariate analysis (in order to achieve the necessary statistical power, EF and CS were excluded). This model showed that APC flow (HR 5.5 CI 1.9–16.2, p = 0.002) was independently associated with the composite outcome, independent of ventricular morphology (HR 0.71 CI 0.30–1.69 p = 0.44) and T1 (HR1.006 CI 1.0–1.-13, p = 0.07).

## Discussion

Fibrotic myocardial remodeling has been identified as an important culprit in the pathogenesis of ventricular dysfunction in acquired heart disease, but only preliminary results on its prevalence, extent, and impact on myocardial function are available for the single ventricle population [8–12]. We present the most comprehensive assessment of myocardial health and cardiac function in Fontan patients.

### Fontan patients demonstrate increased myocardial fibrosis at a young age

LGE has been associated with adverse outcomes in Fontan patients [9]. In our younger Fontan cohort, LGE was less prevalent than previously reported, [9] perhaps reflecting differing surgical strategies or patient demographics. In contrast to LGE, T1 and ECV are markers of diffuse myocardial fibrosis [21]. A small pilot study in Fontan patients found evidence of increased myocardial fibrosis [13]. The present, larger study confirms these findings and links fibrosis markers to worsening myocardial strain and outcome metrics. Furthermore, imaging markers of fibrosis were associated with increased ventricular volumes, worse EF and CS. These observations suggest that diffuse myocardial fibrosis plays a role in the pathogenesis of ventricular dysfunction.

### Adverse outcomes are associated with fibrosis, abnormal myocardial function and volume loading

In the present cohort, patients who experienced the composite outcome had increased fibrosis and decreased myocardial function, evidenced by worse EF and CS. In a series of older Fontan patients, Rathod et al. demonstrated that volume loading was independently associated with death or transplantation [22]. Similarly, in our cohort the amount of APC flow, perhaps mediated through ventricular volume loading, was independently associated with the composite outcome and with higher T1 values [8, 22]. The link between adverse outcomes and APC flow and increased fibrosis should encourage the field to continue to monitor fibrosis longitudinally while investigating management options.

### Right ventricular morphology, increased volume loading and longer cumulative bypass time are potential risk factors for myocardial disease and ventricular dysfunction

Our morphological and functional results add to the growing body of evidence that patients with DRVs have worse ventricular function and, according to some studies, less favorable outcomes [4, 23, 24]. On average, DRV patients had worse EF and myocardial strain, demonstrated a trend towards higher ECV and experience more adverse outcomes than DLV counterparts. Although ventricular morphology was not a significant variable in the Cox regression analysis, it remains unclear whether these observations reflect inherent differences in intrinsic myocardial properties between the right and left ventricles [25, 26], or whether they stem from the more common need for neonatal surgeries, longer cumulative surgical times or an uncaptured variable. While the acute adverse effects of cardiopulmonary bypass on myocardial function are well-known and have resulted in improved myocardial protection [27], the long-term consequences have not been explored in detail. In our series, increased cumulative exposure to cardiopulmonary bypass, aortic cross clamp and circulatory arrest were associated with worse long-term myocardial function, a link not been previously recognized in this population. An alternative explanation for this observation is that the duration of cardiopulmonary bypass may be a surrogate for anatomical complexity or preoperative status which, in turn, could predispose to myocardial dysfunction. Irrespective of the pathophysiology, our observations emphasize the need for better strategies of myocardial protection.

### Increased wall and fiber stress are present in Fontan patients and associated with myocardial dysfunction

The Fontan cohort had larger ventricular volumes as well as lower EF and strain in all domains as compared to controls, corroborating previous findings by both echocardiography and CMR [7, 9, 13]. Circumferential strain was reduced in the Fontan cohort, particularly in those with AVVR and even in those with preserved EF. Impaired CS appeared to be a more sensitive predictor of adverse outcomes than EF [28, 29].

More recently, elevated ESWS and ESFS, which result in an imbalance between myocardial oxygen consumption and supply [30], have emerged as risk factors of adverse outcomes in coronary artery disease and chronic heart failure [20, 31, 32]. In our Fontan cohort and in a previous study [7], both ESWS and ESFS were increased. The physiologic and long-term implications of abnormal wall and fibre stress require ongoing exploration.

### Ventriculoarterial coupling is abnormal in Fontan patients

VAC ratio is a novel parameter that can be measured invasively, using pressure volume loops [33], or non-invasively using CMR volumetry and standard blood pressure oscillometric methods [6, 34] or CMR-derived wave intensity parameters [35]. The VAC ratio describes the interplay between the heart and great vessels. A ratio in the normal range describes a state where the stroke volume is transmitted to the arterial system with a small amount of energy loss [36]. In normal physiology, the VAC ratio resides tightly between 0.5 and 1.2 as was the case in our control healthy population [36, 37]. Compared to healthy controls, and similar to a previous report by Godfrey and colleagues, our Fontan cohort had a higher VAC ratio as well as a greater heterogeneity in VAC within their group [6]. An abnormal VAC suggests that Fontan patients spend a greater amount of energy on the same stroke volume. The etiology of the elevated VAC is likely multifactorial: decreased ventricular Ees appears to play a role, as seen in our cohort, as does arterial stiffness from the surgical patch during the Stage 1 Norwood procedure [35]. The relationship between lower Ees and longer cardiopulmonary bypass, circular arrest, and aortic cross clamp times again underscores the importance of effective cardio-protection.

## Limitations

Several study limitations warrant discussion. First, T1 relaxometry was consistently feasible only in the free wall and our assumption that the values are representative of the entire ventricular myocardium may be erroneous. Second, the control cohort was older than the Fontan group; for ethical reasons, younger children could not be recruited for a contrast-enhanced CMR study under general anaesthesia. However, one would expect a greater degree of myocardial fibrosis with increasing age and thus the older age of the control subjects would underestimate the significance of the fibrosis found in the Fontan population. Furthermore, the subgroup of patients with Fontan failure was small and related results need to be substantiated in a larger cohort. Lastly, two different gadolinium agents had been used, with potential impact on ECV. However, the influence of the type of gadolinium has been shown to be small [38].

## Conclusions

Our results point towards a preclinical adverse cardiac remodeling process which is evident even in young and comparatively healthy Fontan patients. A greater degree of aortopulmonary collateral flow is associated with higher myocardial fibrosis markers and adverse outcomes.

## Supplementary Information

**Additional file 1: Figure S1.** Bland-Altman plot for interobserver variability of ECV, with limits of agreement. There was good agreement and no statistically significant bias. **Figure S2.** Bland-Altman plot for interobserver variability of T1 with limits of agreement. There was good agreement and no statistically significant bias. **Figure S3.** Bland-Altman plot for intraobserver variability of T1 with limits of agreement. There was good agreement and no statistically significant bias. **Figure S4.** Bland and Altman plot for intraobserver variability of ECV with limits of agreement. There was good agreement and no statistically significant bias.

## Data Availability

The datasets used and/or analysed during the current study are available from the corresponding author on reasonable request.

## Abbreviations

APC: Aortopulmonary collateral
AVVR: Atrioventricular valvar regurgitation
CMR: Cardiovascular magnetic resonance
CS: Circumferential strain
DLV: Dominant left ventricle
DRV: Dominant right ventricle
EA: Arterial elastance
ECV: Extracellular volume fraction
EDVI: End diastolic volume indexed to body surface area
Ees: End-systolic ventricular elastance
EF: Ejection fraction
ESFS: End systolic fiber stress
ESVI: End systolic volume indexed
ESWS: End systolic wall stress
LGE: Late gadolinium enhancement
LS: Longitudinal strain
RS: Radial strain
VAC: Ventricular arterial coupling
