# Supplementary material for: Adverse fibrosis remodeling and aortopulmonary collateral flow are associated with poor Fontan outcomes
Source: J Cardiovasc Magn Reson. 2021 Nov 15;23:134. doi: 10.1186/s12968-021-00782-9 (PMC8591885; doi:10.1186/s12968-021-00782-9)
Supplement: Supplementary file 1 — Additional file 1: Figure S1. Bland-Altman plot for interobserver variability of ECV, with limits of agreement. There was good agreement and no statistically significant bias. Figure S2. Bland-Altman plot for interobserver variability of T1 with limits of agreement. There was good agreement and no statistically significant bias. Figure S3. Bland-Altman plot for intraobserver variability of T1 with limits of agreement. There was good agreement and no statistically significant bias. Figure S4. Bland and Altman plot for intraobserver variability of ECV with limits of agreement. There was good agreement and no statistically significant bias. [file 12968_2021_782_MOESM1_ESM.docx]

Additional file 1

Arterial elastance (Ea) = MBP .

SV/BSA

End-systolic elastance (Ees) = MBP .

ESV/BSA

Figure S1


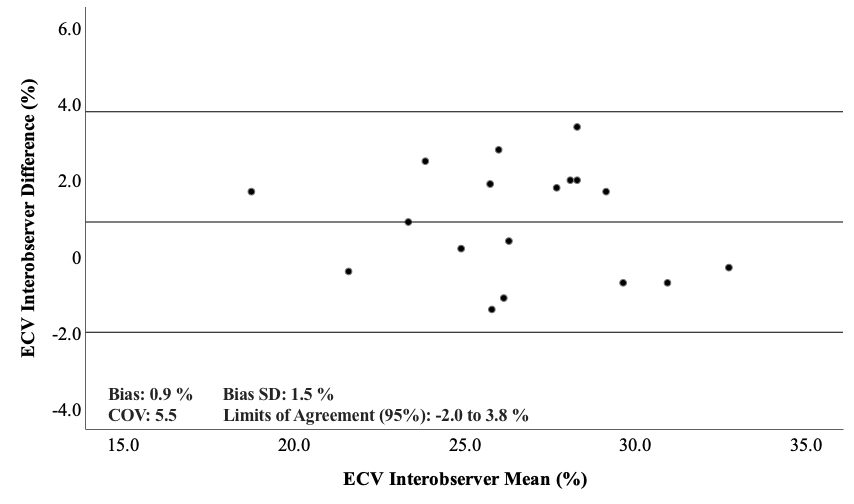


Legend: Bland-Altman plot for interobserver variability of ECV, with limits of agreement. There was good agreement and no statistically significant bias.

Figure S2


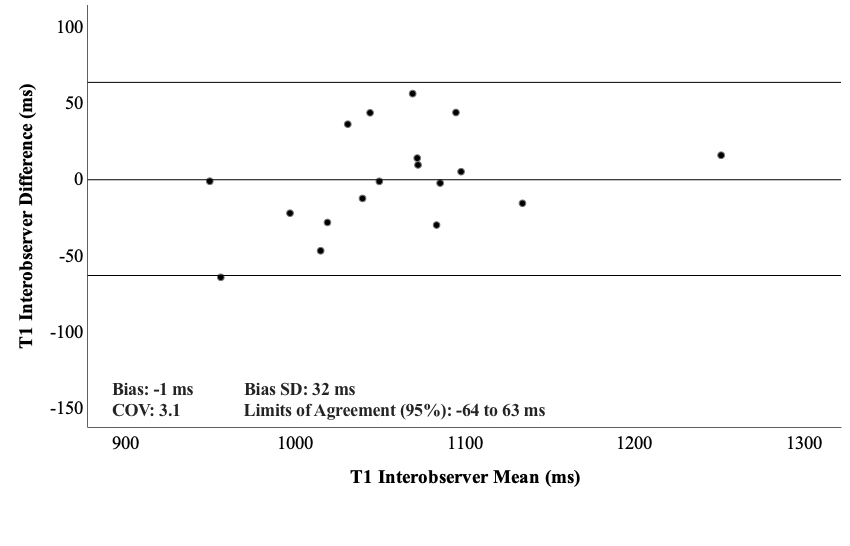


Legend: Bland-Altman plot for interobserver variability of T1 with limits of agreement. There was good agreement and no statistically significant bias.

Figure S3


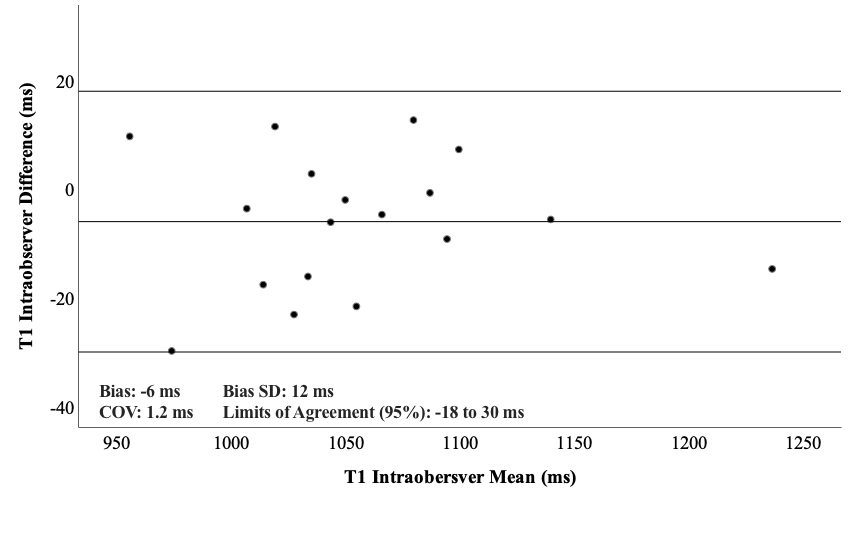


Legend: Bland-Altman plot for intraobserver variability of T1 with limits of agreement. There was good agreement and no statistically significant bias.

Figure S4


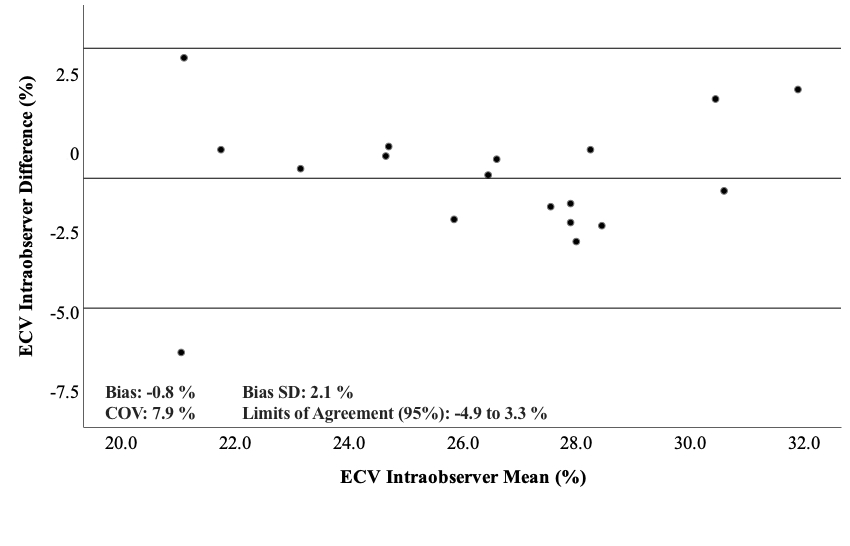


Legend: Bland and Altman plot for intraobserver variability of ECV with limits of agreement. There was good agreement and no statistically significant bias.
